# Photonic integrated circuits and their applications

**DOI:** 10.1093/nsr/nwag233

**Published:** 2026-04-17

**Authors:** Hon Ki Tsang, Jiapeng Luan, Yue Qin, Zelu Wang, Shuqi Xiao, Caiyue Zhao, Liang Zhang, Keyi Zhong, Xuetong Zhou, Yaojing Zhang

**Affiliations:** Department of Electronic Engineering, The Chinese University of Hong Kong, China; Department of Electronic Engineering, The Chinese University of Hong Kong, China; Department of Electronic Engineering, The Chinese University of Hong Kong, China; Department of Electronic Engineering, The Chinese University of Hong Kong, China; Department of Electronic Engineering, The Chinese University of Hong Kong, China; Department of Electronic Engineering, The Chinese University of Hong Kong, China; Department of Electronic Engineering, The Chinese University of Hong Kong, China; Department of Electronic Engineering, The Chinese University of Hong Kong, China; Department of Electronic Engineering, The Chinese University of Hong Kong, China; School of Science and Engineering, The Chinese University of Hong Kong, Shenzhen, China

## Abstract

Perspectives on the future economic impact of the technologies and applications of photonic integrated circuits in AI data interconnects, advanced sensors and quantum computing.

The economic impact of photonic integrated circuits (PICs) is growing significantly in the 21st century. Today, the largest market for PICs is for pluggable optical transceivers, which are now widely deployed in data centers, supporting most of the data traffic on the internet and driving the rise of data-center scale artificial intelligence (AI) compute platforms for training frontier large language models. According to Epoch AI (https://epoch.ai/data-insights/compute-trend-post-2010), the computational requirements of the frontier AI models have grown at a rate of about 4.4 times per year, far exceeding Moore’s law. The computation demands for AI have been met by building scale-up and scale-out networks in data centers. Silicon photonics, which leverages the mature complementary metal-oxide-semiconductor (CMOS) fabrication processes developed for microelectronics, is the only truly scalable platform for high-volume manufacturing, offering the high-density integration needed to support hundreds of data lanes in emerging system architectures. At the ECOC 2025 plenary talk, Edward Lee from NVIDIA asserted that co-packaged optics (CPO) using microring modulators (MRMs), as exemplified in Fig. 1a, with surface input/output (I/O) using grating coupler (GC) arrays, will become the key enabling technologies for scaling up networks needed in large AI compute systems. While the bandwidth density of die-to-die electrical interconnects can exceed 20 Tbps/mm (https://awavesemi.com/silicon-ip/phy-ip/die-to-die-phy-ip/), the minimum pitch of standard single-mode fiber arrays is 127 μm, limiting the number of optical fibers that can be attached per millimeter of chip to about 8. This results in an optical bandwidth density below 1.6 Tbps/mm/λ when using optical edge-couplers (assuming 100 Gbaud PAM 4 modulation). Thus, for high bandwidth density optical I/O used for scale-up networks with much larger clusters of dies, waveguide GCs, rather than traditional edge couplers, may be a preferred solution for CPO, such as the optical transmitter used in optical interconnects in Fig. 1b. The performance of manufacturable waveguide GCs has seen major advances, with less than 0.9 dB coupling loss achievable at 170 nm minimum feature sizes using shifted polysilicon overlay technology [1]. Further improvements in coupling efficiency and 1 dB bandwidth over 200 nm are achievable with advanced technology nodes and designs [2]. However, significant challenges remain for the industry to establish new multi-source agreement standards for pluggable fiber interfaces for use with GC arrays. And if such standards can be established, it could lead to CPO becoming the mainstream replacement of pluggable transceivers in the 2030–2035 timeframe.

**Figure 1.**
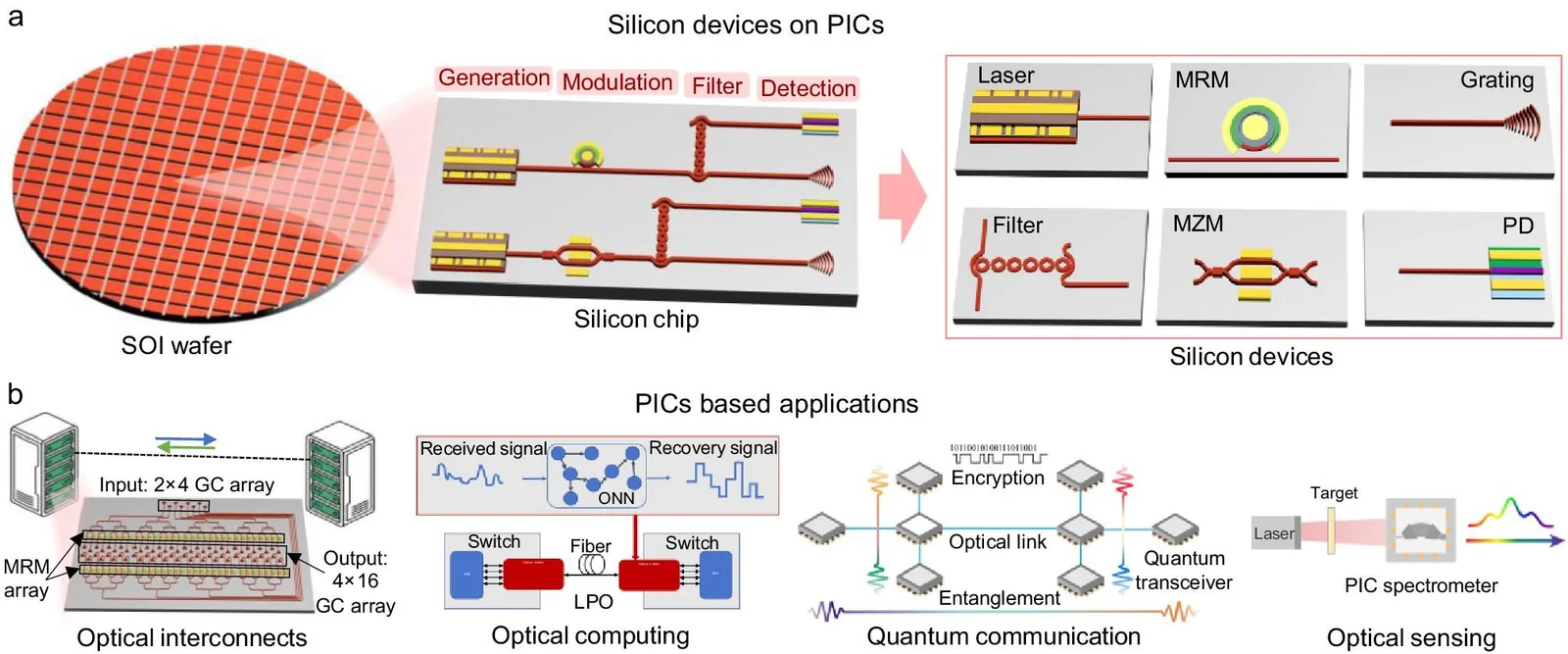
PICs and their applications. (a) Silicon chips fabricated based on silicon-on-insulator (SOI) wafers typically involve light generation, modulation, filtering, and detection from lasers, modulators, filters, and gratings/photodetectors (PDs), respectively. (b) PICs-based applications in optical interconnects, optical computing, quantum communication, and optical sensing.

In the context of CPO, the demand for high integration density comes from the need to have hundreds of optical modulators in each CPO unit. Compact modulator architectures such as MRMs and electro-absorption modulators are favored over the larger footprint Mach-Zehnder modulators. Silicon MRMs have been shown to achieve modulation bandwidths exceeding ∼110 GHz [3], enabling 200 Gbaud per lane net data rate in 400 Gb/s intensity modulation/direct detection links [4]. To achieve the scaling of hundreds of MRM per die, it is also essential to develop robust electrical control of the individual thermal heater to lock the resonances of each MRM to guarantee operation over a wide range of temperatures, and wafer-scale trimming to minimize the power consumption of the wavelength locking circuits. Modulation beyond 400 Gb/s will need the development of modulators with other materials, such as lithium niobate or III-V semiconductors on the silicon platform, and the scaling of these technologies for the high-yield manufacture of the millions of units needed for CPO will be a future challenge. High-speed silicon photodetectors integrated with germanium, group III–V materials, or two-dimensional materials, can operate at frequencies up to 110 GHz, enabling the detection of high-speed signals [1]. An alternative approach to scaling to higher bandwidth density is wavelength-division multiplexing (WDM) technology, which employs microring resonators with large free spectral ranges to support a large number of wavelength channels per fiber. Utilizing low-cost uncooled lasers, coarse WDM systems can achieve low insertion loss and crosstalk while maintaining high fabrication tolerance in the receivers with the use of angled multi-mode interferences as wavelength demultiplexers [1].

In the next few years, pluggable transceivers will remain the dominant technology of choice for hyperscale datacenters. The emerging development of linear pluggable optics (LPO), which reduces the power consumption of traditional transceivers by removing the digital signal processor (DSP) from the optical transceiver, introduces new research challenges on how to maintain high-quality signal transmission beyond 100 Gbaud. Photonic integration of optical signal processors is an emerging research direction to enable LPO transceivers to attain higher data rates and to facilitate the implementation of optical neural networks (ONNs) and high-throughput optical convolution processors. LPOs will be increasingly used in horizontally scaled networks for distributed computing (such as AI training), where the lower latency can improve performance, while the higher-bandwidth interconnects needed to sustain the continued growth of data center traffic will need the lower power consumption possible with LPO. While traditional DSPs can compensate for channel impairments, this comes at the cost of high power consumption as data rates scale to beyond 100 Gbaud. However, implementing ONNs in Fig. 1b, such as a photonic reservoir computing architecture, offers a transformative solution [5]. ONNs implemented on PICs are a promising emerging approach for replacing DSPs in pluggable transceivers and high-speed optical communication systems in the future. Furthermore, ONNs that leverage the speed and parallelism of light can enable high computational density and trainable chip-scale implementation. By integrating arrayed waveguide grating (AWG) convolution engines, end-to-end ONNs can provide high-performance, low-latency processing capabilities for next-generation edge intelligence systems, offering potential in fields such as autonomous systems, health monitoring, and environmental sensing [6].

Beyond data interconnects and optical signal processing, PICs are finding other important emerging applications in quantum information processing and optical sensing. Quantum entangled states have a nonclassical correlation in which the states of entangled particles remain strongly correlated regardless of their spatial separation. Silicon phonics can generate, manipulate, and measure the correlation of entangled photons. Such controllable entangled photons provide key resources for encryption applications, like secure key distribution. Recently, a fully silicon-chip-based integrated quantum transceiver with on-chip quantum state measurement has been demonstrated for long-distance quantum entanglement distribution over 160 km, which is potentially applicable for secure key distribution networks [7]. High-resolution and broadband integrated photonic spectrometers based on tandem AWGs and microring resonators offer a solution for highly portable solid-state and high-speed spectral domain optical coherence tomography systems [8]. Multimode silicon photonics also offers interesting solutions for high-Q cavities for integrated nonlinear photonic systems [9]. Heterogeneous integration of III–V group materials using aspect ratio trapping is also a promising technology for the monolithic integration of on-chip laser sources while maintaining compatibility with CMOS technology for mass production, thereby enabling fully integrated photonic systems [10].

In summary, silicon photonics is a key enabling technology for data centers and AI infrastructures, and is developing as a fundamental and widely used platform technology for many different applications. Currently, its economic value is primarily reflected in data center interconnects, supporting the expansion of AI and cloud computing. However, in the long term, its significance lies in its broad cross-domain applications. This field is progressing along a clear development path characterized by the pursuit of ultimate performance (such as bandwidth and energy efficiency) and deep integration at the device and system levels. Among these, ONNs based on PICs hold the promise of replacing traditional DSPs, achieving lower power consumption and lower latency signal processing in pluggable transceivers and high-speed optical communications. This represents not only a breakthrough in performance but also an evolution in system architecture. The future of PICs extends far beyond high-speed communications as indicated in Fig. 1b. Through deep integration with adjacent fields such as materials science and quantum physics, silicon photonics is becoming central to a series of new system paradigms, encompassing cutting-edge directions such as optical sensing, scalable quantum processing, and even ultra-high-speed optical computing. The evolution from discrete photonic components to fully integrated chip photonic systems may mark a key juncture, making PICs one of the core engines of technological innovation in this century.
